# Lrp5 and Lrp6 Exert Overlapping Functions in Osteoblasts during Postnatal Bone Acquisition

**DOI:** 10.1371/journal.pone.0063323

**Published:** 2013-05-10

**Authors:** Ryan C. Riddle, Cassandra R. Diegel, Julie M. Leslie, Kyle K. Van Koevering, Marie-Claude Faugere, Thomas L. Clemens, Bart O. Williams

**Affiliations:** 1 Department of Orthopaedic Surgery, Johns Hopkins University School of Medicine, Baltimore, Maryland, United States of America; 2 Veterans Administration Medical Center, Baltimore, Maryland, United States of America; 3 Center for Skeletal Disease and Tumor Metastasis and Laboratory of Cell Signaling and Carcinogenesis, Van Andel Research Institute, Grand Rapids, Michigan, United States of America; 4 Department of Medicine, University of Kentucky, Lexington, Kentucky, United States of America; INSERM U1059/LBTO, Université Jean Monnet, France

## Abstract

The canonical Wnt signaling pathway is critical for skeletal development and maintenance, but the precise roles of the individual Wnt co-receptors, Lrp5 and Lrp6, that enable Wnt signals to be transmitted in osteoblasts remain controversial. In these studies, we used Cre-loxP recombination, in which Cre-expression is driven by the human osteocalcin promoter, to determine the individual contributions of Lrp5 and Lrp6 in postnatal bone acquisition and osteoblast function. Mice selectively lacking either Lrp5 or Lrp6 in mature osteoblasts were born at the expected Mendelian frequency but demonstrated significant reductions in whole-body bone mineral density. Bone architecture measured by microCT revealed that Lrp6 mutant mice failed to accumulate normal amounts of trabecular bone. By contrast, Lrp5 mutants had normal trabecular bone volume at 8 weeks of age, but with age, these mice also exhibited trabecular bone loss. Both mutants also exhibited significant alterations in cortical bone structure. *In vitro* differentiation was impaired in both Lrp5 and Lrp6 null osteoblasts as indexed by alkaline phosphatase and Alizarin red staining, but the defect was more pronounced in Lrp6 mutant cells. Mice lacking both Wnt co-receptors developed severe osteopenia similar to that observed previously in mice lacking β-catenin in osteoblasts. Likewise, calvarial cells doubly deficient for Lrp5 and Lrp6 failed to form osteoblasts when cultured in osteogenic media, but instead attained a chondrocyte-like phenotype. These results indicate that expression of both Lrp5 and Lrp6 are required within mature osteoblasts for normal postnatal bone development.

## Introduction

The canonical Wnt signaling pathway is a key regulator of the development of many tissues, but the specific biological roles of the Wnt co-receptors low-density lipoprotein receptor-related protein 5 (Lrp5) and Lrp6 in the skeleton remain controversial. Wnt ligands bind to a receptor complex containing a member of the Frizzled family of receptors and either Lrp5 or Lrp6. These cell surface events trigger a cascade of intracellular reactions that facilitate nuclear import of β-catenin, which then interacts with the Lef/Tcf class of DNA binding proteins to regulate target gene expression [1], [2].

Lrp5 and Lrp6 are 71% homologous and form a distinct subfamily of the LDL receptor-related proteins [3]–[5]. Both co-receptors have four YWTD-β-propeller domains that are followed by EGF-like domains and allow binding to extracellular ligands, and five intracellular PPP(S/T)P domains that mediate downstream signaling events [1]. Previous studies have suggested that Lrp5 and Lrp6 mediate distinct actions owing to differences in tissue distribution and affinity for individual Wnt ligands [6]–[8]. For example, in the developing embryo Lrp6 is widely expressed and homozygosity for a germline inactivating allele results in neonatal lethality, suggesting that this co-receptor plays a dominant role in early development [9]. The expression pattern of Lrp5 is more restricted [5], [10], [11].

All components of the canonical Wnt signaling pathway, including Lrp5 and Lrp6, are expressed by the osteoblast [12]–[14], and have been firmly linked with bone development and maintenance in mice and humans. The importance of LRP5 in bone was initially established by demonstrating that inactivating mutations in this co-receptor caused osteoporosis pseudoglioma (OPPG), a rare syndrome associated with premature and generalized osteoporosis [13]. Subsequently, patients carrying a point mutation in LRP5 (G171V) that increases Wnt signaling capacity were shown to exhibit strikingly high bone mass [15], [16]. Individuals with mutations in LRP6 also develop osteoporosis and display other abnormalities including diabetes, hypertension, and early-onset cardiovascular disease associated with high levels of LDL and triglycerides [17].

While the importance of canonical Wnt signaling in skeletal development is well documented [18], [19], the sequence of molecular events that activate this pathway both up- and downstream of β-catenin are less well defined. In particular, the precise roles of Lrp5 and Lrp6 remain controversial, despite the compelling observations made in humans. For example mice globally deficient for Lrp5 are born without discernable defects in the axial or appendicular skeleton but acquire low bone mass postnatally [14], [20]–[22], which was attributed to a direct effect of Lrp5 on osteoblast function. However, recent studies suggested that the effect of Lrp5 on bone acquisition is attributable to alterations in duodenal serotonin production [23]–[25], although other studies suggest that Lrp5 acts within osteoblasts to regulate bone mass [26], [27]. By contrast, Lrp6−/− mice display defects in both limb and axial development [9]. In addition, analysis of mice heterozygous for a germline inactivating allele or homozygous for a hypomorphic allele of Lrp6 have found they display lower bone mass [7], [22], [28], [29].

In this study, we created mouse models that permit us to clarify the roles of Lrp5 and Lrp6 in bone during postnatal life. Our studies indicate that expression of both Wnt co-receptors is required within osteoblasts for normal skeletal homeostasis.

## Methods

### Generation of Transgenic Mice

The Institutional Animal Care and Use Committees of the Johns Hopkins University (Animal Welfare Assurance #A3272-01; protocol MO11M273) and the Van Andel Research Institute (Animal Welfare Assurance #A4383-01; protocol 10-04-015) approved all animal procedures. Animals were monitored on a daily basis by trained staff members of the animal care facilities and any animals showing signs of illness or distress were humanely euthanized to minimize suffering. Euthanasia of animals for distress or the required timepoints was carried via inhalation of isoflurane followed by cervical dislocation in accordance with the recommendations of the 2000 American Veterinary Medical Association Panel Report on Euthanasia. Lrp5^flox^ and Lrp6^flox^ mice have been described previously [26], [30]. To disrupt the expression each gene in the osteoblasts, floxed mice were crossed with Oc-Cre mice, in which cre recombinase is expressed with high specificity and penetrance in the mature osteoblasts [31]. Genotyping strategies are available upon request. Mice were maintained on a mixed background of C57Bl/6J, 129 and FVB/N. β-catenin^flox^ mice [32] were obtained from Jackson Laboratories.

### Bone Mineral Density Analysis by DXA

Mice were anesthetized via inhalation of 2% isoflurane (TW Medical Veterinary Supply) with oxygen (1.0 L/min) for 10 min prior to imaging and during the procedure (≤ 5 min). The mice were placed on a specimen tray in a PIXImus II bone densitometer (GE Lunar) for analysis. Bone mineral density was calculated by the PIXImus software based on the active bone area in the subcranial region within the total body image.

### Microcomputed Tomography (microCT)

High resolution images of the mouse femur were acquired using a desktop microtomographic imaging system (Skyscan 1172, Skyscan, Belgium) in accordance with the recommendations of the American Society for Bone and Mineral Research (ASBMR) [33]. The femur was scanned at 50 keV and 200 µA using a 0.5 mm aluminum filter with an isotropic voxel size of 10 µm. The resulting two-dimensional cross-sectional images are shown in gray scale. Cortical bone parameters were assessed at the femoral midshaft and represent an average of 50 CT slices (500 µm). Trabecular bone parameters were assessed in the distal femur 500 µm proximal to the growth plate and extending for 2 mm (200 CT slices).

### Bone Histology

Dynamic bone formation was assessed at 8 weeks of age by injection of two sequential.25 ml doses of calcein (0.8 mg/ml) delivered 3 and 8 days prior to sacrifice. The femur was fixed in ethanol, dehydrated and embedded in methylmethacrylate. Three micron sections were cut with a Microm microtome and stained with Mason-Goldner trichrome stain. The number of osteoblasts and osteoclasts per bone perimeter were measured at standardized sites under the growth plate at a magnification of 200X using a semi-automatic method (Osteoplan II, Kontron). These parameters comply with the guidelines of the nomenclature committee of the ASBMR [34].

### Osteoblast Isolation and Adenovirus Infection

Osteoblasts were isolated from calvaria of newborn Lrp5^flox/flox^, Lrp6^flox/flox^, Lrp5^flox/flox^;Lrp6^flox/flox^, and β-catenin^flox/flox^ mice by serial digestion in a 1.8 mg/ml collagenase type I (Worthington Biochemical) solution. Calvaria were digested in 10 ml of digestion solution for 15 min at 37°C with constant agitation. The digestion solution was collected and the digest repeated an additional 4 times. Digestion solutions 3–5, containing osteoblasts, were pooled and cultured in α-MEM supplemented with 10% FBS and 1% penicillin/streptomycin at 37°C in a humidified incubator supplied with 5% CO_2_. To disrupt Lrp5, Lrp6, or β-catenin expression *in vitro*, osteoblasts were grown to approximately 70% confluence and then infected with control adenovirus expressing green-fluorescent protein (ad-GFP) or adenovirus expressing Cre recombinase (ad-CRE, Vector Biolabs) at a MOI of 100. Osteoblasts were harvested 48 h after adenoviral infection and deletion efficiency was assessed in a portion of the cell population by qPCR. The remaining cells were re-plated for proliferation and differentiation assays.

### Osteoblast Proliferation and Differentiation

Osteoblast proliferation was assessed by flow cytometry. Briefly, control, ΔLrp5, and ΔLrp6 osteoblasts were cultured in α-MEM containing 1% FBS for 48 h. BrdU (10 µM) was added to the medium for the last 24 h before harvesting the cells. Cells were stained with anti-BrdU-APC and 7-amino-actinomycin D and analyzed by FACSCAlibur (BD Biosciences). Twenty thousand events were collected for each sample and the results analyzed by WinMDI version 2.8. For differentiation experiments, control, ΔLrp5, ΔLrp6, ΔLrp5ΔLrp6, and Δβ-catenin osteoblasts were grown to confluence and then cultured for 14 days in the presence of 10 mM β-glycerol phosphate and 50 µg/ml ascorbic acid. Fixed cells were stained with 1-Step NBT/BCIP (Pierce) to examine alkaline phosphatase activity. Mineralization was assessed using Alizarin Red (40 mM) and the von Kossa method (3% AgNO_3_). Alcian Blue staining (1% solution in acetic acid) was used to detect proteoglycan deposition.

### Quantitative Real-time PCR

Total RNA was extracted from bone tissue or osteoblasts grown *in vitro* using the Trizol method (Invitrogen). One microgram of pure RNA was reverse transcribed using the iScript cDNA synthesis system (Bio-rad). Two microliters of cDNA was then subjected to PCR amplification using iQ SYBR Green Supermix (Bio-rad). Primer sequences were obtained from PrimerBank (http://pga.mgh.harvard.edu/primerbank/index.html). Reactions were normalized to endogenous β-actin reference transcript.

### Statistical Analysis

Results are expressed as mean ± SEM. All statistical tests were two-sided. A p-value less that 0.05 was considered significant. Comparability of two groups of data was assessed using a Students *t*-test.

## Results

### ΔLrp5 Mice Acquire an Osteopenic Phenotype

**Figure 1:**
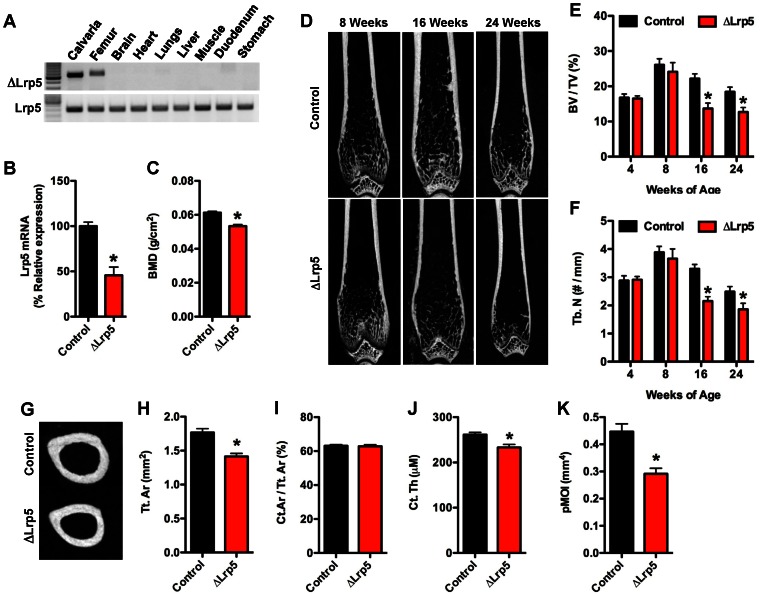
ΔLrp5 mice develop an osteopenic phenotype. (A) PCR analysis of Cre-mediated recombination of the Lrp5^flox^ allele in ΔLrp5 mice. Note the recombination (upper panel) is only evident in skeletal tissue. (B) qPCR analysis of Lrp5 mRNA levels in the femur of control and ΔLrp5 mice. (C) Whole-bone mineral density assessed by DXA at 6months of age (n = 16–18 mice). (D) Representative microCT images of bone structure in the distal femur of control and ΔLrp5 mice at 8, 16, and 24 weeks of age. (E) Quantification of trabecular bone volume per tissue volume (BV/TV) in the distal femur. (F) Quantification of trabecular numbers (Tb.N) in the distal femur. (G) Representative microCT images of cortical bone structure at the femoral mid-diaphysis in 24 weeks old control and ΔLrp5 mice. (H) Cortical tissue area (Tt.Ar). (I) Cortical bone area per tissue area (Ct.Ar/Tt.Ar). (J) Cortical thickness (Ct. Th). (K) Polar moment of inertia (pMOI). For microCT analyses, n = 5–7mice/genotype. *p<0.05.

To begin to characterize the functions of Lrp5 and Lrp6 in bone development, we first generated mice in which Lrp5 was selectively disrupted in the osteoblast. Male progeny from matings between Oc-Cre-Tg+; Lrp5^flox/flox^ and Lrp5^flox/flox^ [26], [30] mice with the genotype Oc-Cre-Tg+; Lrp5^flox/flox^ (hereafter referred to as ΔLrp5) were selected for detailed analysis. ΔLrp5 mice were born at the expected Mendelian ratios, had a normal lifespan, and allele specific PCR confirmed that recombination occurred only in skeletal tissue (ie calvaria and femur, Figure 1A). Littermates lacking the Oc-Cre transgene (Lrp5^flox/flox^) served as controls.

ΔLrp5 mice exhibited a 55% reduction in Lrp5 expression levels in mRNA extracted from intact femurs when compared to control littermates (Figure 1B), and the disruption of this gene resulted in a significant reduction in whole-body bone mineral density (BMD) when examined by DXA at 6 months of age (Figure 1C). The magnitude of the decrease in BMD in ΔLrp5 mice was similar to that reported in mice globally deficient for Lrp5 [21], [22], suggesting that the actions of Lrp5 in osteoblasts directly regulate bone mass.

To examine the structural changes in bone that led to a reduction in BMD in ΔLrp5 mice, we performed a longitudinal analysis of bone architecture using microCT (Figure 1D–F). Assessment of trabecular bone volume in the distal femur at both 4 and 8 weeks of age revealed that disrupting Lrp5 in the osteoblast did not impact the attainment of peak trabecular bone volume. However, by 16 weeks of age ΔLrp5 mice exhibited significant reductions in both trabecular bone volume (37%, Figure 1E) and trabecular numbers (36%, Figure 1F). These deficiencies in trabecular bone structure persisted through 24 weeks of age and were accompanied by significant alterations in cortical bone architecture (Figure 1G–K). At this time point, cortical tissue area was reduced by approximately 20% in ΔLrp5 mice relative to controls (Figure 1H), while cortical thickness was reduced by approximately 10% (Figure 1J). As a result, the polar moment of inertia was reduced by more than 34% in the mutant mice when compared to controls (Figure IK).

Quantitative histomorphometry performed at the distal femur at 8 weeks confirmed that trabecular bone volume was normal in Lrp5 mutant (data not shown), but suggested that the bone phenotype was beginning to develop at this time point (Table 1). ΔLrp5 mice had normal numbers of osteoblasts and osteoclasts. However, the mineralizing surface per bone surface was reduced by more that 61% in ΔLrp5 mice when compared to controls and was accompanied by a decrease in the bone formation rate and an increase in mineralization lag time. Taken together these findings suggest that the loss of Lrp5 function in osteoblasts can be compensated for during the period of rapid bone growth prior to 8 weeks of age, but Lrp5 is required for proper osteoblast function later in postnatal life.

**Table 1:** Bone histomorphometry.

Bone parameter	Control	ΔLrp5
Bone formation		
Osteoblast number/bone perimeter(NOb/BPm; no./100 mm)	267.60±104.06	175.68±29.54
Osteoid surface/bone surface (OS/BS; %)	6.76±1.61	5.12±0.55
Osteoid thickness (O.Th; µm)	1.75±0.18	1.98±0.15
Bone Erosion		
Osteoclast number/bone perimeter(NOc/BPm; no./100 mm)	290.96±26.08	296.50±25.76
Erosion surface/bone surface (ES/BS; %)	7.20±0.37	7.71±1.12
Erosion Depth (EDE, µm)	5.36±0.34	6.17±0.55
Bone Dynamics		
Mineral apposition rate (MAR; µm/day)	3.16±0.17	2.84±0.18
Mineralizing surface/bone surface(MS/BS; %)	13.71±1.84	5.26±0.39*
Bone formation rate/bone surface(BFR/BS; mm^3^/cm^2^/yr)	15.55±1.85	5.46±0.56*
Mineralization lag time (Mlt; day)	0.32±0.06	0.88±0.14*

Values are shown as mean ± S.E. (n = 5–6 per genotype at 8 weeks of age).

* p<0.05.

### ΔLrp6 Mice Fail to Accumulate Trabecular Bone

**Figure 2:**
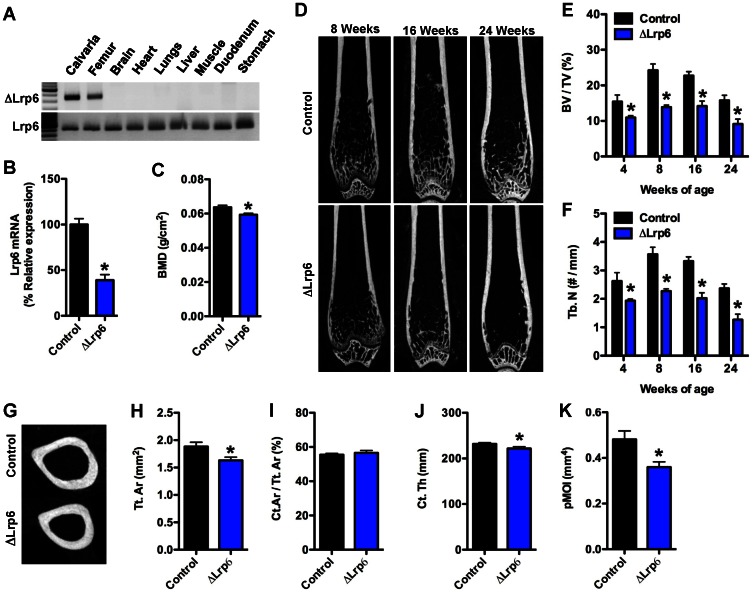
Trabecular and cortical bone acquisition is reduced in ΔLrp6 mice. (A) PCR analysis of Cre-mediated recombination of the Lrp6^flox^ allele in ΔLrp6 mice. (B) qPCR analysis of Lrp6 mRNA levels in the femur of control and ΔLrp6 mice. (C) Whole-bone mineral density assessed by DXA at 6months of age (n = 16–26 mice). (D) Representative microCT images of bone structure in the distal femur of control and ΔLrp6 mice at 8, 16, and 24 weeks of age. (E) Quantification of trabecular bone volume per tissue volume (BV/TV) in the distal femur. (F) Quantification of trabecular numbers (Tb.N) in the distal femur. (G) Representative microCT images of cortical bone structure at the femoral mid-diaphysis in 24 weeks old control and ΔLrp6 mice. (H) Cortical tissue area (Tt.Ar). (I) Cortical bone area per tissue area (Ct.Ar/Tt.Ar). (J) Cortical thickness (Ct. Th). (K) Polar moment of inertia (pMOI). For microCT analyses, n = 5–8mice/genotype. *p<0.05.

Using an approach identical to that employed to examine Lrp5 function, we next generated mice in which Lrp6 was selectively disrupted in the osteoblast. Oc-Cre-Tg+; Lrp6^flox/flox^ (hereafter referred to as ΔLrp6) mice were born at the expected Mendelian ratios, had a normal lifespan, and PCR confirmed that recombination occurred only in bone (Figure 2A) and resulted in a significant reduction in Lrp6 mRNA (56%, Figure 2B).

**Figure 3:**
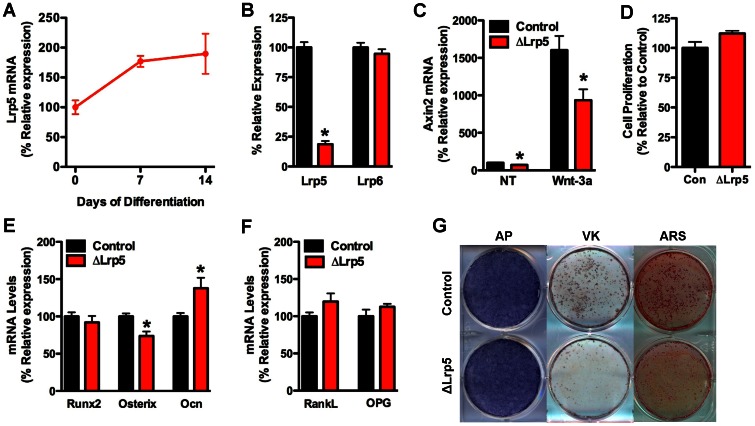
Lrp5 acts in the late stages of osteoblast differentiation. (A) Lrp5 mRNA levels in primary calvarial osteoblasts were assessed by qPCR during 14 days of osteogenic differentiation. (B) Lrp5 and Lrp6 mRNA levels were assessed by qPCR in calvarial osteoblasts isolated from Lrp5 floxed mice and infected with adenovirus expressing GFP (control) or Cre recombinase. (C) Axin2 mRNA levels were examined in control and ΔLrp5 osteoblasts in cells treated with 100 ng/ml Wnt-3a. (D) Cell proliferation was assessed by BrdU incorporation. Osteogenic (E) and osteoclastogenic markers (F) were examined by qPCR after 14 days of differentiation in osteogenic media. (G) Mineralized nodule formation was assessed by staining for alkaline phosphatase (AP), von Kossa (VK) and Alizarin red (ARS) after 14 days of differentiation. *p<0.05.

Much like the genetic ablation of Lrp5, disruption of Lrp6 in osteoblasts resulted in a significant reduction in whole-body BMD when examined by DXA at 6 months of age (Figure 2C), but deficiencies in bone structure were evident much earlier in these mutants. Whereas ΔLrp5 mice exhibited normal trabecular bone volume until 8 weeks of age, deficiencies in trabecular architecture were evident as early as 4 weeks of age in ΔLrp6 mice when compared to control littermates (Figure 3D–F). Moreover, ΔLrp6 mice failed to achieve peak trabecular bone volume and exhibited a 42% decrease in trabecular bone volume at 8 weeks of age (Figure 3E). This phenotype was accompanied by a profound decrease in the number of osteoblasts and significant reductions in the mineral apposition rate and bone formation rate (Table 2).

**Table 2:** Bone histomorphometry.

Bone parameter	Control	ΔLrp6
Bone formation		
Osteoblast number/bone perimeter(NOb/BPm; no./100 mm)	1383.94±332.58	496.77±81.65*
Osteoid surface/bone surface (OS/BS; %)	20.96±4.10	10.59±1.77*
Osteoid thickness (O.Th; µm)	1.69±0.25	1.46±0.24
Bone Erosion		
Osteoclast number/bone perimeter(NOc/BPm; no./100 mm)	277.42±74.09	245.63±56.25
Erosion surface/bone surface(ES/BS; %)	6.28±1.02	5.38±1.01
Erosion Depth (EDE, µm)	5.12±0.39	6.17±0.39
Bone Dynamics		
Mineral apposition rate (MAR; µm/day)	3.67±0.26	2.97±0.14*
Mineralizing surface/bone surface(MS/BS; %)	23.85±5.31	13.21±2.02
Bone formation rate/bone surface(BFR/BS; mm^3^/cm^2^/yr)	27.57±5.12	14.29±2.09*
Mineralization lag time (Mlt; day)	0.43±0.13	0.53±0.15

Values are shown as mean ± S.E. (n = 5–6 per genotype at 8 weeks of age).

* p<0.05.

Cortical bone structure was also influenced by the loss of Lrp6 function in osteoblasts (Figure 2G–K). When compared to control littermates at 24 weeks of age, cortical tissue area and cortical thickness were reduced by 13% and 4%, respectively, in the ΔLrp6 mice. As a result, the polar moment of inertia was reduced by 25% in the mutant mice. These data imply that Lrp6 is required earlier in postnatal bone development than Lrp5.

### Disrupting Lrp5 or Lrp6 Impairs Osteoblast Differentiation *in vitro*

**Figure 4:**
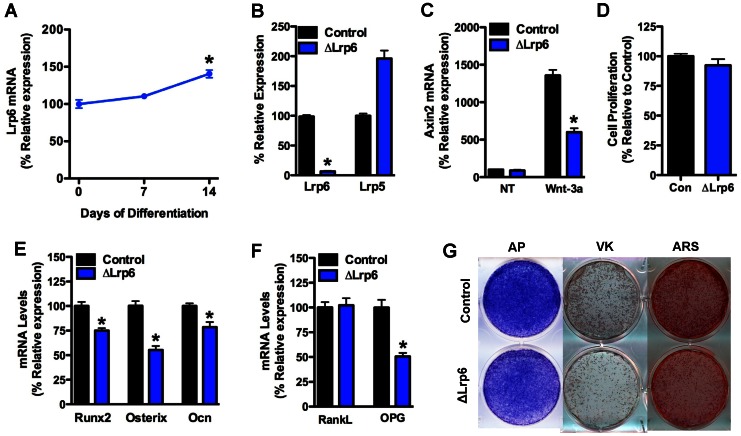
Loss of Lrp6 impairs osteoblast differentiation. (A) Lrp6 mRNA levels in primary calvarial osteoblasts were assessed by qPCR during 14 days of osteogenic differentiation. (B) Lrp6 and Lrp5 mRNA levels were assessed by qPCR in calvarial osteoblasts isolated from Lrp6 floxed mice and infected with adenovirus expressing GFP (control) or Cre recombinase. (C) Axin2 mRNA levels were examined in control and ΔLrp6 osteoblasts in cells treated with 100 ng/ml Wnt-3a. (D) Cell proliferation was assessed by BrdU incorporation. Osteogenic (E) and osteoclastogenic markers (F) were examined by qPCR after 14 days of differentiation in osteogenic media. (G) Mineralized nodule formation was assessed by staining for alkaline phosphatase (AP), von Kossa (VK) and Alizarin red (ARS) after 14 days of differentiation. *p<0.05.

To characterize the cellular basis for the disturbances in bone development observed in the mutant mice, we examined the expression of each Wnt co-receptor during the differentiation of primary osteoblasts. Lrp5 and Lrp6 mRNA were both expressed in osteoblasts but the relative levels varied during the course of differentiation. Lrp5 mRNA expression increased during the differentiation process such that Lrp5 mRNA levels at day 14 were 53% higher than at day 0 (Figure 3A). By contrast, the expression of Lrp6 remained stable during the first 7 days of differentiation, but increased by approximately 40% at day 14 (Figure 4A).

We next tested the effect of selectively disrupting each of the co-receptors on osteoblast performance *in vitro*. Calvarial osteoblasts were isolated from the floxed mice and adenoviral-Cre was introduced to disrupt the Lrp genes. Disrupting the expression of Lrp5 had no effect on Lrp6 mRNA (Figure 3B), but significantly decreased Wnt3a-induced expression of Axin2 (Figure 3C), indicating the requirement for Lrp5 in canonical Wnt signaling. Osteoblasts deficient for Lrp5 proliferated normally (Figure 3D), expressed alkaline phosphatase (Figure 3G) and exhibited only minimal alterations in the expression of markers of osteoblast differentiation (Figure 3E and 3F). However, matrix mineralization, assessed by Alizarin red and von Kossa staining, was markedly impaired relative to controls (Figure 3G).

Disruption of Lrp6 in primary osteoblasts (Figure 4B) also impaired Wnt signaling as indexed by reduced Wnt3a-induced Axin2 expression (Figure 4C). The loss of Lrp6 resulted in a more profound defect in osteoblast differentiation, even though these cells expressed markedly increased levels of Lrp5 mRNA (Figure 4B). Lrp6-deficient osteoblasts proliferated normally (Figure 4D), but markers of differentiation including the expression of Runx2, Osterix and Osteocalcin were all decreased by approximately 30% in 14 day cultures (Figure 4E). These changes in gene expression were accompanied by profound decreases in alkaline phosphatase staining and mineralized nodule formation (Figure 4G). Notably, the expression of OPG was reduced by nearly 50% (Figure 4F) in accordance with previous studies that demonstrate regulation of OPG by Wnt-induced β-catenin signaling [18], [19]. Taken together, these *in vitro* data suggest that Lrp5 and Lrp6 function at distinct times during osteoblast differentiation.

### Osteoblast Lrp5/Lrp6 Double Mutants Develop Severe Osteopenia

**Figure 5:**
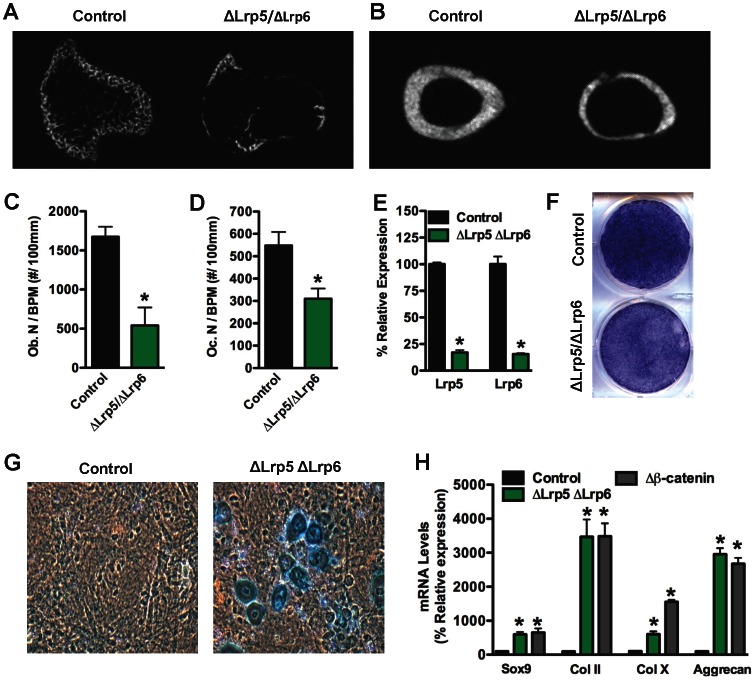
ΔLrp5/Lrp6 double mutants exhibit severe bone loss. Representative microCT images from the proximal tibia (A) and femoral midshaft (B) are shown for 4 week old control and OBΔLrp6/ΔLrp6 mice. (C) Osteoblasts numbers per bone perimeter (Ob.N/BPM) and (D) Osteoclasts numbers per bone perimeter (Oc.N/BPM) were quantified in the distal femur of 4 week old mice. (E) Lrp5 and Lrp6 mRNA levels were assessed by qPCR in calvarial osteoblasts isolated from Lrp5/Lrp6 floxed mice and infected with adenovirus espressing GFP (control) or Cre recombinase. (F) Osteogenic differentiation was assessed by staining for alkaline phosphatase after 14 days. Alcian blue staining (G) and qPCR (H) were used to identify cells with a chondrocyte-like phenotype in cultures of control, ΔLrp5/ΔLrp6, and Δβ-catenin calvarial cells grown under osteogenic conditions.

To explore the possibility of functional redundancy of Lrp5 and Lrp6 in bone acquisition, we analyzed mice that lacked both Lrp5 and Lrp6 in osteoblasts (ΔLrp5/ΔLrp6) by crossing the two individual mutants. Double mutant mice were born at the expected Mendelian frequency but less than 50% survived beyond 7 weeks of age and fewer than 10% survived beyond 14 weeks. Examination of bones from 4 week old animals indicated severe loss of both trabecular and cortical bone (Figure 5A and 5B), a phenotype indistinguishable from that observed in mice lacking β-catenin in the osteoblast [18]. At this time point, osteoblasts numbers were reduced by more that 66%, while the number of osteoclasts was reduced by 45% (Figure 5C and 5D).

Finally, we determined the impact of disrupting both Lrp5 and Lrp6 in primary osteoblasts (Figure 5E). Osteoblasts lacking both Wnt co-receptors proliferated normally (data not shown), but demonstrated greatly impaired differentiation as assessed by reductions in alkaline phosphatase staining (Figure 5F). Moreover, a significant portion of cells in the 14-day cultures stained positively for Alcian Blue and exhibited morphological and other features compatible with chondrocytes (Figure 5G), including increased expression of the chondrocyte differentiation markers Sox9, Collagen II, Collagen X and Aggrecan (Figure 5H). A similar effect was observed in primary calvarial cells lacking β-catenin. Thus the combined loss of both Lrp5 and Lrp6 in osteoblasts appears to diminish or eliminate Wnt signaling which in turn diverts the allocation of cells from the osteoblast to chondrocyte lineage.

## Discussion

Recent studies have documented the importance of canonical Wnt signaling in skeletal development and postnatal bone acquisition [18], [19]. However, the precise sequence of molecular events that control signaling activity through this pathway both up- and downstream of β-catenin are still unclear. In particular, the relative contribution of the Wnt co-receptors Lrp5 and Lrp6 to canonical Wnt signaling in bone has been controversial, in part because of differences in the experimental models used to study this pathway [13], [14], [21]–[24]. Here we used a genetic approach in mice, which enabled us to unequivocally determine the impact of disruption of Lrp5 and Lrp6 in osteoblasts during postnatal bone acquisition. Our results demonstrate that both co-receptors are required for normal postnatal bone acquisition, but suggest that Lrp5 and Lrp6 exert selective actions in the osteoblast.

At birth and through 8 weeks of age, ΔLrp5 mice were grossly normal and had no observable alterations in bone structure. These findings accord with observations made in mice globally deficient for Lrp5, which have normal bone mass at birth [14], and indicate that the skeletal defects in mammals with Lrp5 dysfunction manifest primarily during the postnatal period. The notion that bone abnormalities are an acquired consequence of Lrp5 dysfunction was also suggested by Yadav *et al* [23], [24], who attributed the loss of bone mass in Lrp5 null mice to alterations in serotonin production by the intestine. In this study, osteoblast-specific disruption of Lrp5 as a result of Cre expression driven by the type I collagen promoter did not alter vertebral trabecular bone volume, bone formation rate, osteoblast numbers, or the expression of genes associated with osteoblast differentiation. The discrepancy between our results and those reported by Yadav [23], [24], could be the result of differences in genetic background, the skeletal site examined, or the time at which bone structure was examined. Indeed, our data suggest that the bone phenotype in ΔLrp5 mice is just beginning to develop at 8 weeks of age, as parameters of mineralization (MS/BS, BFR/BS and MLT) were significantly altered but microCT and histological analyses revealed trabecular bone volume to be normal.

The developmental abnormalities and skeletal defects in mice globally deficient in Lrp6 are more severe than those of Lrp5 mutants; Lrp6 mutants die in the immediate postnatal period with loss of distal limb structures and truncation of the axial skeleton [9]. This suggests that Lrp6 may function to a greater degree during embryogenesis and likely impacts skeletal development at earlier stages. In agreement with this idea, primary osteoblasts lacking Lrp6 exhibited a more profound defect in differentiation than those lacking Lrp5. Moreover, the ΔLrp6 mice demonstrated reductions in trabecular bone volume as early as 4 weeks of age and did not achieve peak bone volume. Together these data suggest that Lrp5 and Lrp6 function to control Wnt/β-catenin activity over different windows of time during skeletal development. Additional studies to determine the effect of deleting each of these co-receptors at selected times and in different osteoblast lineages would be required to test this idea more definitively.

A noteworthy finding from our study was the observation that both Lrp5 and Lrp6 in osteoblasts influenced cortical bone architecture. ΔLrp5 mice exhibit the alterations in tissue cross-sectional area that have also been reported in Lrp5 null mice [21] and the effect of manipulating this co-receptor appeared to be more severe than the effect of disrupting Lrp6. Since the trabecular bone phenotype was more severe in ΔLrp6 mice, these data suggest that relative importance of Lrp5 and Lrp6 may differ across bone envelopes. Nonetheless, our data indicates a need to explore the roles of Lrp6 as well as Lrp5 in skeletal mechanotransduction [21], [35].

Despite differences in genetic background, histomorphometric measurements and the results of our *in vitro* studies provide a mechanistic explanation for the differences observed in the two mutants. ΔLrp5 mice had normal numbers of osteoblasts but exhibited reduced bone formation rates and increased mineralization lag times. Likewise, primary osteoblasts lacking Lrp5 were able to produce alkaline phosphatase at levels similar to controls but failed to form mineralized nodules normally. These defects in mineralization together with the pattern of Lrp5 expression during osteoblast differentiation in vitro suggest that this Wnt co-receptor functions in the late stages of osteoblast differentiation. The loss of Lrp5 function during this stage of osteoblast differentiation could more easily be compensated for during early postnatal skeletal development when the numbers of osteoblasts are high. Osteoblasts deficient for Lrp6 differentiated poorly *in vitro*, producing lower levels of alkaline phosphatase and exhibiting reduced levels of osteogenic gene expression, and ΔLrp6 mice exhibited dramatically reduced numbers of osteoblasts. These data indicate a defect early in the differentiation process of the osteoblast. Considering the marked reduction in matrix mineralization in Lrp6 deficient osteoblasts *in vitro* and reduced numbers of osteoblasts *in vivo*, we were surprised that dynamic measures of osteoblast activity including mineral apposition rates and mineralization lag time were not affected to a greater degree in ΔLrp6 mice. This observation may result from the marked up-regulation of Lrp5 mRNA following the deletion of Lrp6. Consistent with this idea, mice expressing a mutant Lrp5 receptor that enhances Wnt signaling in osteoblasts display dramatically increased parameters of mineralization [36].

The more dramatic effect of Lrp6 loss of function on bone volume may also be related to the influence of osteoblasts on osteoclast development. Lrp6 deficient osteoblasts expressed lower levels of OPG, which is consistent with the notion that canonical Wnt signaling regulates the coupling of osteoblasts and osteoclasts [19]. That the numbers of osteoclasts in ΔLrp6 were comparable to those in controls, must be viewed in the context of the dramatic reduction in osteoblasts in the mutants. In this regard the ratio of osteoclasts to osteoblasts would be much higher in the mutant mice than in the controls.

Despite the differences in the skeletal phenotypes in ΔLrp5 and ΔLrp6 mice, it is evident that the Wnt co-receptors exert overlapping functions. Wnt-3a-induced expression of Axin2 was diminished but not abolished by the disruption of either Wnt co-receptor and only disrupting both Lrp5 and Lrp6 in osteoblasts produced a bone phenotype reminiscent of that observed in mice lacking β-catenin in these cells [18]. These double mutants exhibited severely reduced trabecular and cortical bone and less than 10% survived beyond 14 weeks of age. Osteoblasts lacking both Lrp5 and Lrp6 demonstrated greatly impaired osteoblast differentiation and attained a chondrocyte like phenotype in agreement with previous studies in osteoblasts lacking β-catenin [37]. Thus, the combined loss of both Lrp5 and Lrp6 in osteoblasts appears to be required to fully eliminate Wnt signaling and in turn divert the allocation of cells from the osteoblast to chondrocyte lineage. This is consistent with the deficiency in commitment to the osteoblast lineage seen in embryos in which Lrp5 and Lrp6 were deleted from osteochondral progenitors using Dermo1-cre [26].

In summary, our studies demonstrate that the highly related Wnt co-receptors Lrp5 and Lrp6 are expressed in osteoblasts and are both required for normal postnatal bone acquisition in mice. We conclude that the activities of these co-receptors regulate canonical Wnt signaling in osteoblasts over different windows of time and in different skeletal compartments to promote proper acquisition of both cortical and trabecular bone.
